# Comments on ‘*BAF60a-dependent chromatin remodeling preserves β-cell function and contributes to the therapeutic benefits of GLP-1R agonists*’

**DOI:** 10.1093/jmcb/mjaf044

**Published:** 2025-11-25

**Authors:** Qingqian Wu, Yue Gao, Zhuo-Xian Meng

**Affiliations:** Zhejiang Key Laboratory of Traditional Chinese Medicine for the Prevention and Treatment of Senile Chronic Diseases, Department of Geriatrics, Hangzhou First People’s Hospital, Hangzhou 310006, China; Department of Pathology and Pathophysiology and Department of Hepatobiliary and Pancreatic Surgery of the Second Affiliated Hospital, Zhejiang University School of Medicine, Hangzhou 310058, China; Zhejiang Key Laboratory of Traditional Chinese Medicine for the Prevention and Treatment of Senile Chronic Diseases, Department of Geriatrics, Hangzhou First People’s Hospital, Hangzhou 310006, China; Zhejiang Key Laboratory of Traditional Chinese Medicine for the Prevention and Treatment of Senile Chronic Diseases, Department of Geriatrics, Hangzhou First People’s Hospital, Hangzhou 310006, China; Department of Pathology and Pathophysiology and Department of Hepatobiliary and Pancreatic Surgery of the Second Affiliated Hospital, Zhejiang University School of Medicine, Hangzhou 310058, China

Type 2 diabetes (T2D) is a highly prevalent metabolic disease characterized by impaired glucose-stimulated insulin secretion (GSIS) and progressive failure of pancreatic β-cell. However, the underlying molecular mechanisms governing β-cell dysfunction during the course of T2D remain incompletely understood, and current therapeutic strategies show limited and heterogeneous benefits. Maintaining β-cell identity and functional plasticity under metabolic stress is essential for glucose homeostasis. Accumulating evidence indicates that epigenetic remodeling is a central driver of β-cell adaptation and deterioration (Kim and Kulkarni, 2020; Wang et al., 2022). Among epigenetic regulators, ATP-dependent switch/sucrose non-fermentable (SWI/SNF) complexes have emerged as key determinants of β-cell transcriptional competence. However, whether individual SWI/SNF subunits act as metabolic sensors in β-cells is still unclear.

In our recent work (Qiu et al., 2025), we performed transposase-accessible chromatin using sequencing (ATAC-Seq) analysis of human and mouse diabetic β-cells and identified widespread alterations in chromatin accessibility, particularly at distal enhancer-like regions that regulate insulin secretion and glucose metabolism, consistent with previous reports (Rai et al., 2020). This enhancer-centered remodeling prompted us to search for upstream chromatin regulators, and through integrated multi-omics coupled with functional screening, we identified BAF60a as a key SWI/SNF subunit required for maintaining β-cell GSIS under metabolic stress. Lipotoxic and inflammatory stimuli markedly reduced BAF60a expression, and β-cell-specific deletion of BAF60a impaired insulin secretion and decreased the expression of β-cell identity genes, whereas restoration of BAF60a expression rescued these defects. Mechanistically, BAF60a cooperates with the β-cell transcription factor (TF) Nkx6.1 to direct transcriptional programs that are essential for glucose metabolism and insulin secretion.

A naturally occurring BAF60a mutation (V278M) in humans was associated with impaired GSIS in human islets, and mice carrying this variant recapitulated β-cell dysfunction, indicating a clinically relevant role for BAF60a in human β-cell physiology. There is an urgent need to determine whether additional human variants in BAF60a or its cofactors contribute to β-cell failure through similar mechanisms. Previous clinical studies reported significant inter-individual variability in response to GLP-1 receptor (GLP-1R) agonists (Dawed et al., 2023). Our findings suggest that BAF60a regulates the expression of incretin receptors, including *Glp1r* and *Gipr*, thereby influencing the insulinotropic action of GLP-1R agonists such as semaglutide. These results raise the possibility that inter-individual variation in chromatin remodeling capacity may contribute to heterogeneous therapeutic responses among individuals.

**Figure 1:**
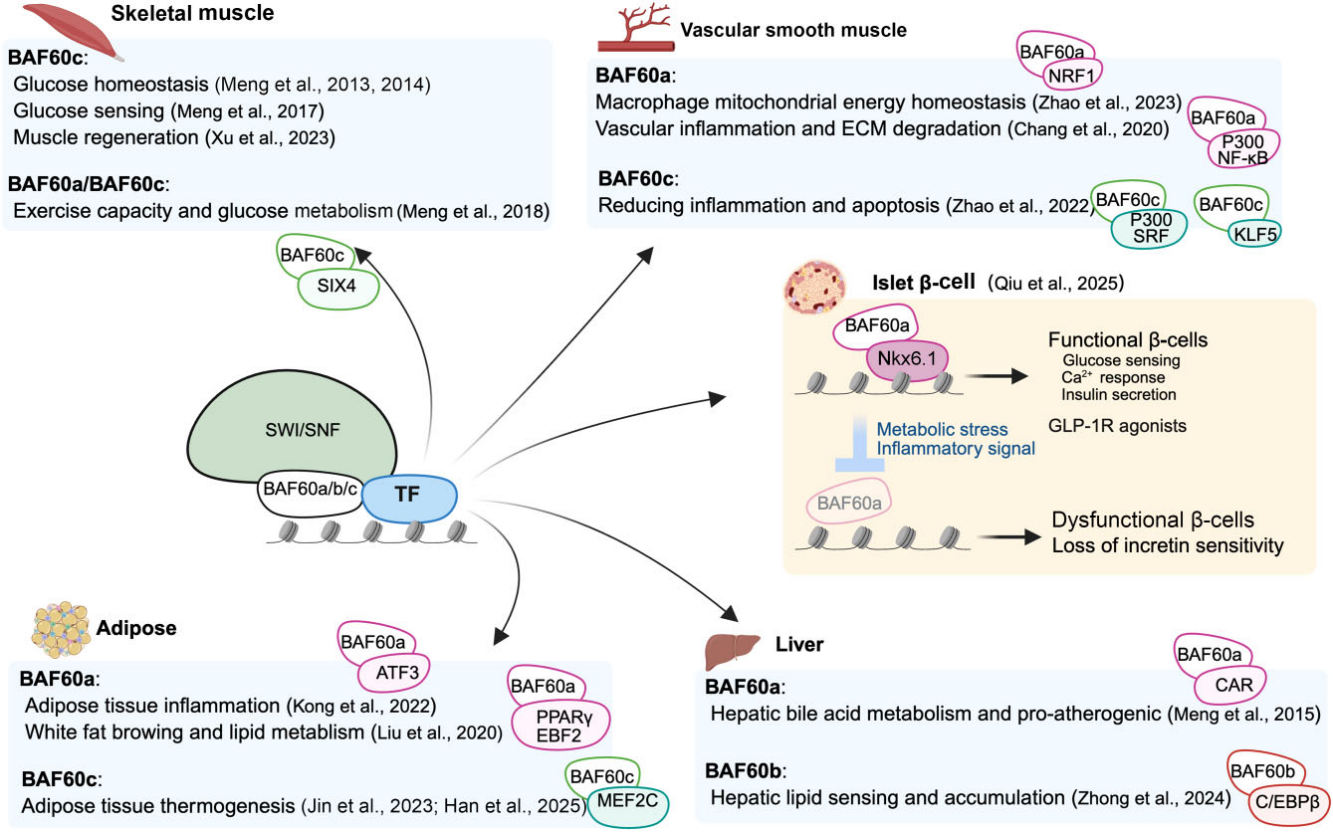
Summary of past studies defining tissue-specific metabolic functions of the BAF60 family (created with BioRender.com).

Over the past decade, our team has systematically delineated the tissue-specific functions of BAF60 family members within the SWI/SNF chromatin remodeling complex (Figure 1). In skeletal muscle, our earlier studies established that BAF60c acts as a key driver of glycolytic reprogramming and systemic glucose homeostasis (Meng et al., 2013, 2014) and further demonstrated that combined BAF60a and BAF60c deficiency disrupts the metabolic coupling between muscle activity and whole-body homeostasis (Meng et al., 2018). Crucially, subsequent work expanded this framework by identifying essential roles of BAF60c in glucose sensing and muscle regeneration (Meng et al., 2017; Xu et al., 2023). Beyond skeletal muscle, accumulating work has highlighted the diverse roles of BAF60 subunits in adipose and hepatic metabolism. Myeloid BAF60a functions as a critical checkpoint that constrains obesity-associated adipose tissue inflammation (Kong et al., 2022), In parallel, BAF60a has been implicated in promoting white fat browning and coordinating lipid metabolic remodeling (Liu et al., 2020). Moreover, two independent lines of evidence demonstrated that BAF60-dependent signaling plays an essential role in adipose tissue thermogenesis, supporting beige fat oxidative capacity and systemic energy balance (Jin et al., 2023; Han et al., 2025). Extending beyond adipose biology, BAF60b was shown to modulate lipid homeostasis through its interaction with C/EBPβ (Zhong et al., 2024), while BAF60a engages a constitutive androstane

receptor (CAR)-dependent hepatic program that drives bile acid and cholesterol metabolism and exerts a pro-atherogenic effect (Meng et al., 2015). Further supporting the versatility of the BAF60 family, studies in abdominal aortic aneurysm models revealed that BAF60a promotes aneurysm development by enhancing inflammatory activation and compromising extracellular matrix (ECM) stability in vascular smooth muscle cells, whereas BAF60c plays a protective role by maintaining the contractile phenotype and reducing inflammation and apoptosis to preserve smooth muscle homeostasis (Chang et al., 2020; Zhao et al., 2022). In addition, myeloid BAF60a deficiency was shown to accelerate atherosclerosis by compromising macrophage and mitochondria homeostasis (Zhao et al., 2023). Collectively, these studies provide an integrated view of how the BAF60 family confers tissue-specific metabolic functions across muscle, immune, and adipose systems and vasculature.

In conclusion, our study identifies BAF60a as a key chromatin regulator that integrates metabolic signals with enhancer remodeling and β-cell transcriptional programs during T2D progression, thereby preserving β-cell functional competence. Several promising directions for future research warrant further exploration. Investigating whether loss of BAF60a alters GLP-1R downstream signaling could provide insight into inter-individual variability in incretin responsiveness. Single-cell and spatial multi-omics approaches may help delineate how BAF60a regulates distinct β-cell subpopulations under metabolic stress. In addition, assessing whether pharmacological or gene-based enhancement of BAF60a activity can improve β-cell sensitivity to GLP-1R agonists represents a promising avenue for translational research.

*[This work was supported in part by grants from the National Natural Science Foundation of China (82425012, 92457301, and 32471170), the National Science and Technology Major Project of Cancer, Cardiovascular and Cerebrovascular Diseases, Respiratory Diseases and Metabolic Diseases Prevention and Treatment Research (2025ZD0550400), Zhejiang Provincial Natural Science Foundation (LHDMD24H030001), and the Construction Fund of Key Medical Disciplines of Hangzhou (2025HZZD06).]*
